# Supplementary material for: A Novel Metastatic Estrogen Receptor-Expressing Breast Cancer Model with Antiestrogen Responsiveness
Source: Cancers (Basel). 2023 Dec 9;15(24):5773. doi: 10.3390/cancers15245773 (PMC10742098; doi:10.3390/cancers15245773)
Supplement: Supplementary file 1 [file cancers-15-05773-s001.zip › cancers-2548894-supplementary.pdf]

Supplementary Figure Document

Supplemental Table S1. Primary antibodies used for immunohistochemistry of primary tumors and bone.

Antibody	Concentration	Company	Catalog No.	RRID
ERα	1:800	Millipore Sigma	06-935	AB_310305
ERα	1:50	Thermo Fisher	sc-8005	AB_10986080
Src	1:1000	Cell Signaling Technology	2108S	AB_331137
CD3	1:750	Abcam	Ab16669	AB_443425
CD4	1:100	Cell Signaling Technology	25229S	AB_2798898
CD8a	1:200	Cell Signaling Technology	98941S	AB_2756376
CD68	1:2000	Abcam	Ab125212	AB_10975465
CD45r (B220)	1:500	Abcam	Ab64100	AB_1140036
Neutrophil elastase	1:500	Abcam	Ab68672	AB_1658868
Pan-cytokeratin	1:1000	Cell Signaling Technology	4545S	AB_490860
Sca-1	1:400	Abcam	Ab109211	AB_10862573
Endomucin	1:200	ThermoFisher Scientific	14-5851-82	AB_891527

	Average Heart Weight (g ± SEM)	Average Lung Weight (g ± SEM)	Average Kidney Weight (g ± SEM)	Average Liver Weight (g ± SEM)	Average Spleen Weight (g ± SEM)
**ER+ 4T1.2**	0.14 ± 0.01	0.25 ± 0.01	0.31 ± 0.01	1.21 ± 0.03	0.64 ± 0.04
**TN 4T1.2**	0.16 ± 0.01	0.26 ± 0.02	0.30 ± 0.01	1.24 ± 0.06	0.79 ± 0.08

Supplemental Table S2. There are no differences in organ weights between mice with ER+ and TN 4T1.2 tumors. Organ weights (heart, lung, kidney, liver, and spleen) were collected at necropsy. There were no significant differences in the weights of organs between animals with different tumor types ($p>0.05$, Mann-Whitney or Student's t-test).

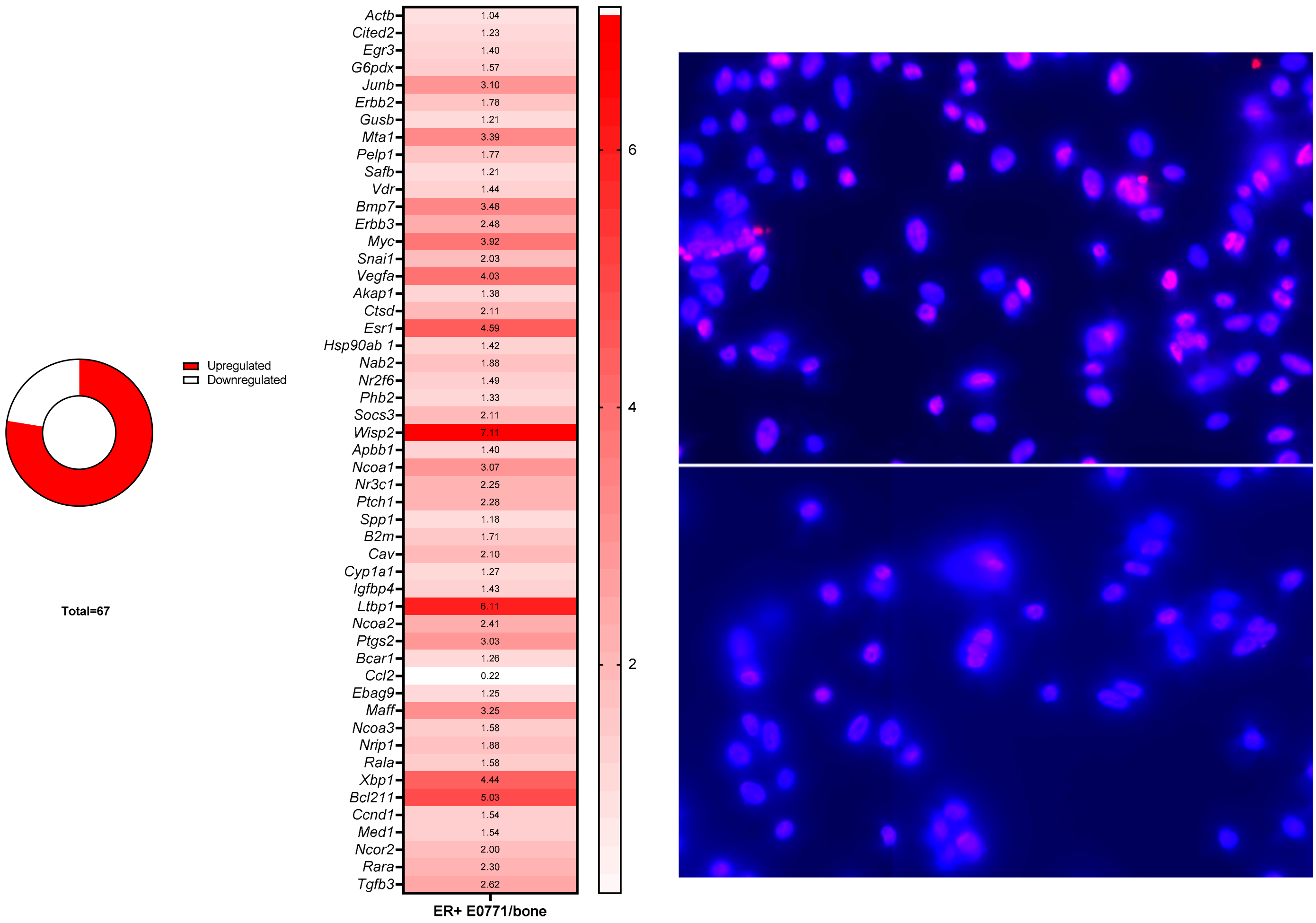

Supplemental Figure S1. ER+ E0771/bone cells displayed high expression of genes associated with estrogen signaling. **A.** RNA was collected from ER+ and TN E0771/bone cells when the cells reached 70% confluence. PCR was performed and normalized to the housekeeping gene *Gapdh* and compared between ER+ and TN E0771/bone cells. Of the 67 genes identified on the PCR array, 52 were upregulated in the ER+ E0771/bone cells compared to the TN E0771/bone cells. The upregulated genes are expressed as fold change of ER+ over TN. **B.** There is more ER expression (red) in

ER+ E0771/bone cells (top) compared with TN E0771/bone cells (bottom). DAPI was used as a nuclear stain (blue). Images were taken at 10X.

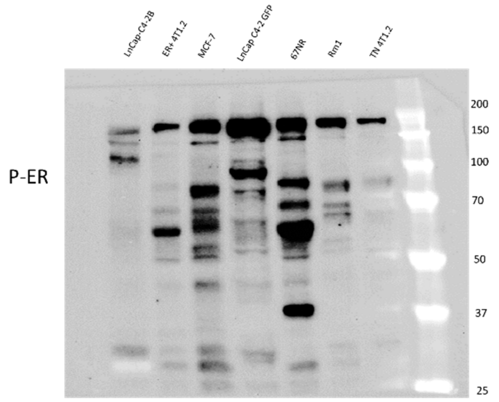

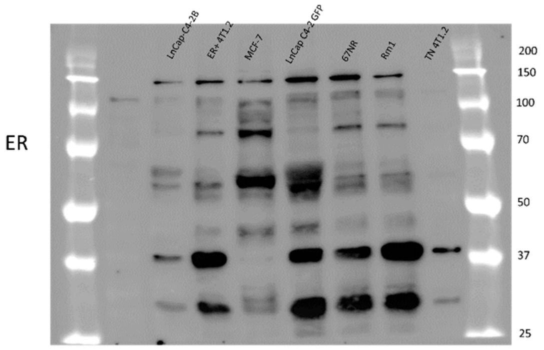

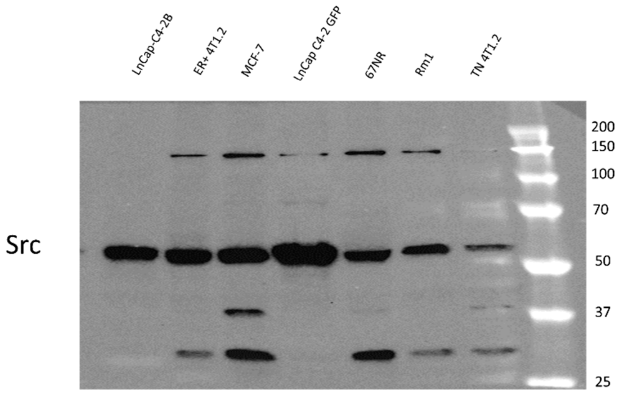

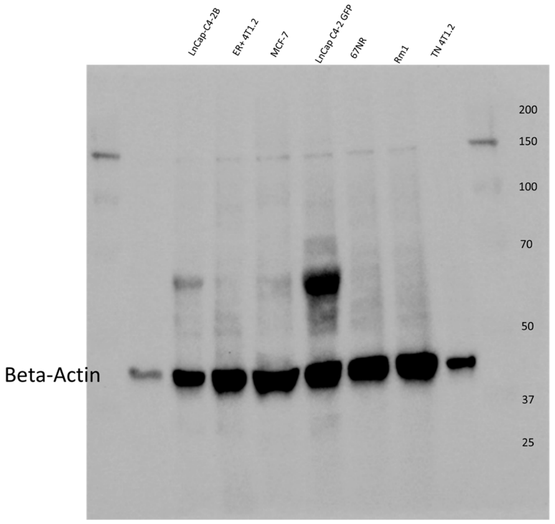

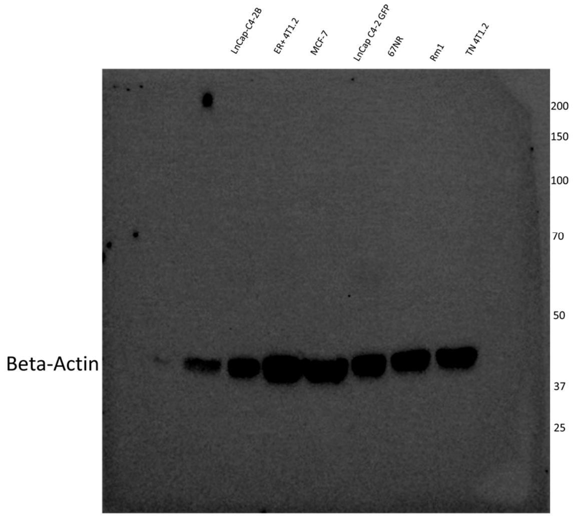

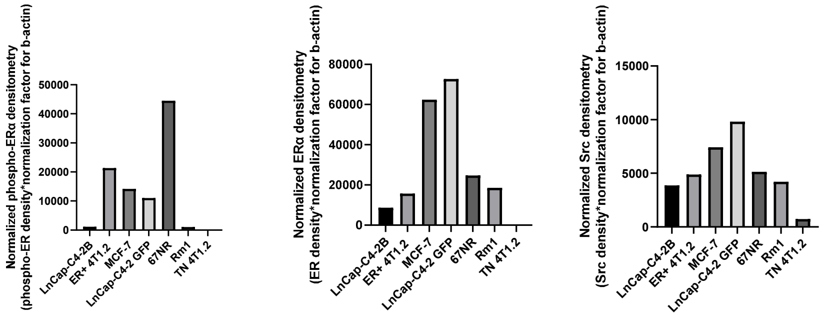

Supplemental Figure S2. Immunoblots with densitometry for ER, phosphor-ER, SRC, and B-actin. Full, original blots for the immunoblots in **Figure 1c**, the first B-actin was used to normalize the y-ER and ER blots, the second B-actin was used to normalize the SRC blot. Densitometry for the western blots plotted as band intensity of each sample by antibody, divided by the normalization factor of the corresponding housekeeping protein (b-actin).

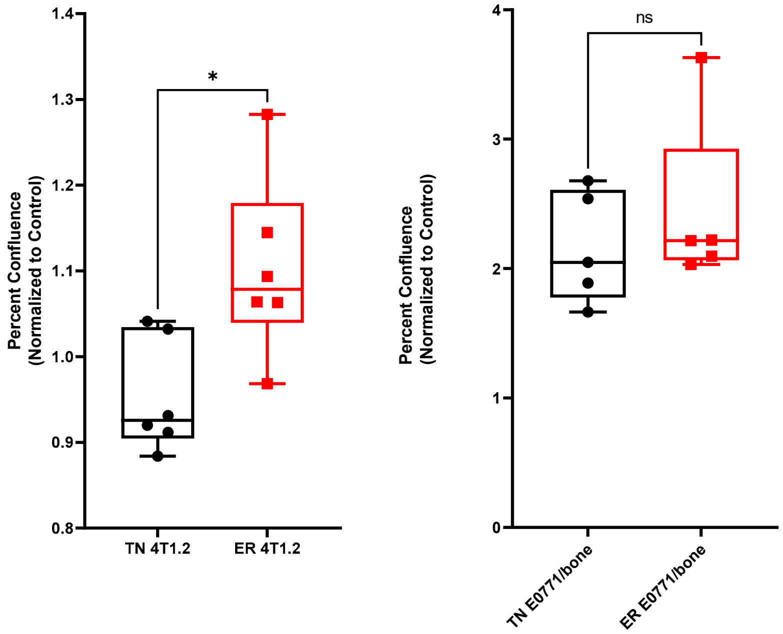

Supplemental Figure S3. ER expressing 4T1.2 cells have increased proliferation in estrogen rich environments compared with TN 4T1.2 (normalized to estrogen-low environment). 4T1.2 and E0771/bone cells were grown in either high estrogen (complete media) or estrogen-low media (phenol red-free and charcoal stripped FBS) for 48 hours. At 48 hours, the confluence of cells was calculated using the Incucyte Zoom and the increased growth in estrogen rich environments was calculated for each condition. The ER+ 4T1.2 cells had significantly increased proliferation in estrogen-

rich environments compared with TN 4T1.2. Both are represented as mean percentage ± SEM (n=4–8). * represents p<0.05 by Student's T-test.

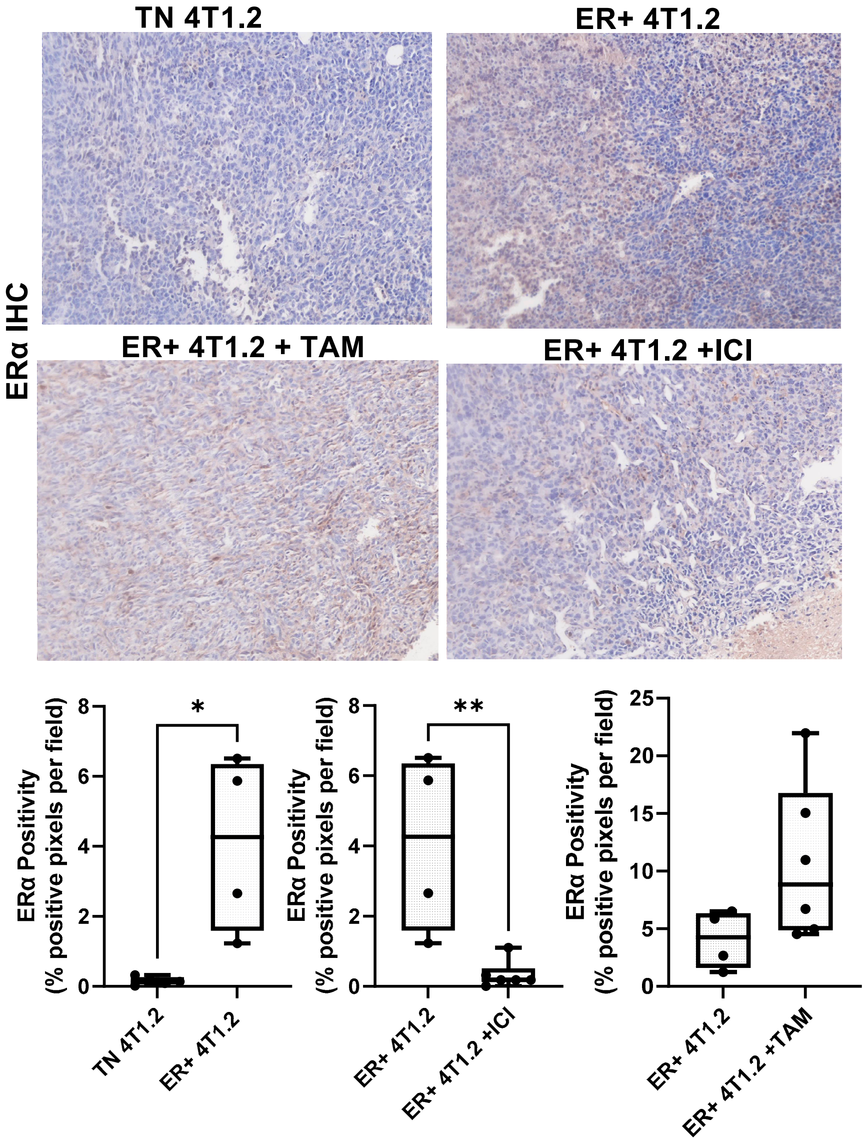

Supplemental Figure S4. Representative example ER IHC in ER expressing and TN 4T1.2 orthotopic tumors. Mice with ER expressing tumors had significantly increased ER expression within the tumors, as determined by IHC with VisioPharm analysis to determine percent positivity. Furthermore, mice treated with ICI had significantly decreased ER expression in the primary tumors. Mice treated with TAM did not have significantly different ER expression compared with non-treated, ER expressing tumors. Both are represented as mean percentage ± SEM (n=4–8). * represents p<0.05, ** represents p<0.01 by Mann-Whitney test.

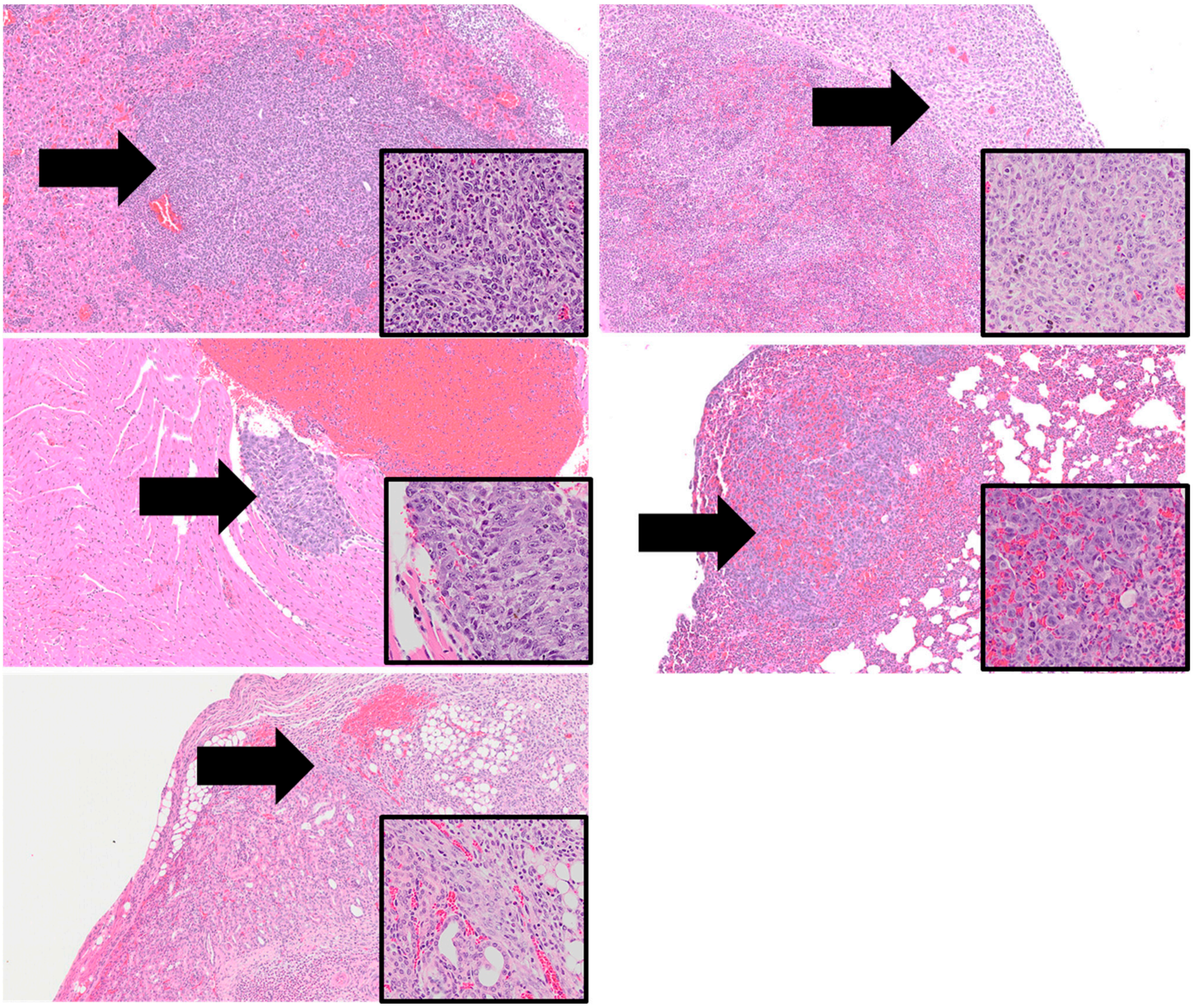

Supplemental Figure S5. Examples of metastasis in major organs (arrow indicating metastasis). The left column show liver, heart, and kidney from top to bottom and the right column shows spleen and lung (all images taken at 5x with a 20x insert).

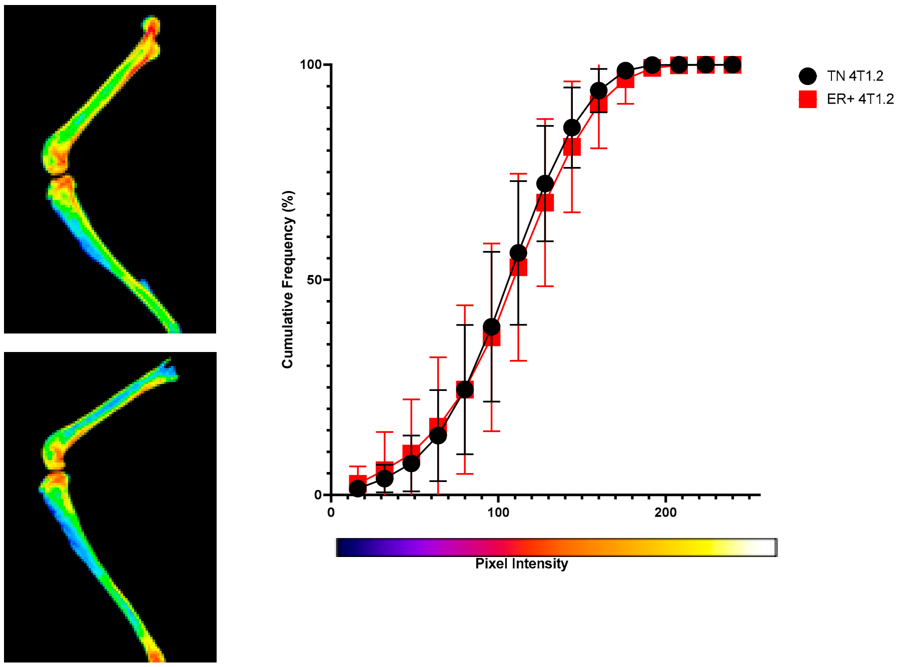

Supplemental Figure S6. There is no significant difference in hind limb mineral density between mice with ER+ and TN 4T1.2 tumors. Hind limbs from mice were collected and soft tissue was removed following tumor implantation with ER+ or TN 4T1.2 cells. Bones were fixed in formalin and radiographs were taken using the Faxitron® imaging system with aluminum and polyester standards which were manually used to set the high/low intensity of the image. Images were scaled to 8-bit formatting, inverted, and pseudocolored with a 16-color scale for ER+ (A) and TN (B) hindlimb radiographs. Frequency of pixel hue was calculated and graphed as cumulative frequency (C). There were no significant differences in the cumulative pixel frequency (mineral density) of the hind limbs from the ER+ and TN bearing mice. Cumulative pixel frequency is represented as mean percentage ± SEM (n=21–23), the p value was calculated using a Kolmogorov-Smirnov test with significance <0.05.

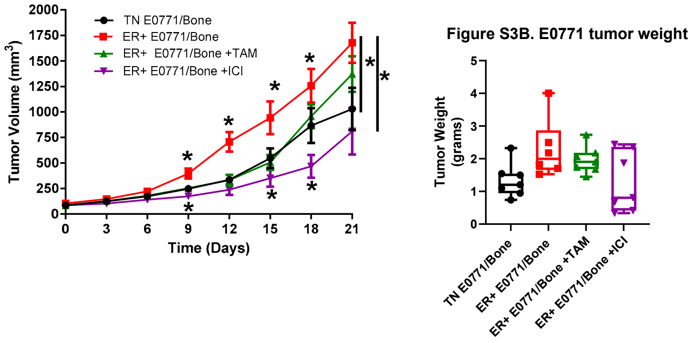

Supplemental Figure S7. ER+ tumors are responsive to antiestrogen treatment. ER+ and TN E0771/bone BC cells were injected into the 4th inguinal mammary fat pad in C57Bl/6 mice. Once tumor volumes reached 100 mm^3, mice were treated with vehicle control, 5 mg/ 60-day time-release pellet TAM, or 1 mg/wk ICI. Tumor volume was

tracked over time and are represented as mean tumor volume ±SEM (n=4–7). Tumor weight was measured upon experimental termination and are represented as mean tumor weight ±SEM (n=4–7). * represents p<0.05 and *** represents p<0.005 by two-way (A) or one-way ANOVA (B).

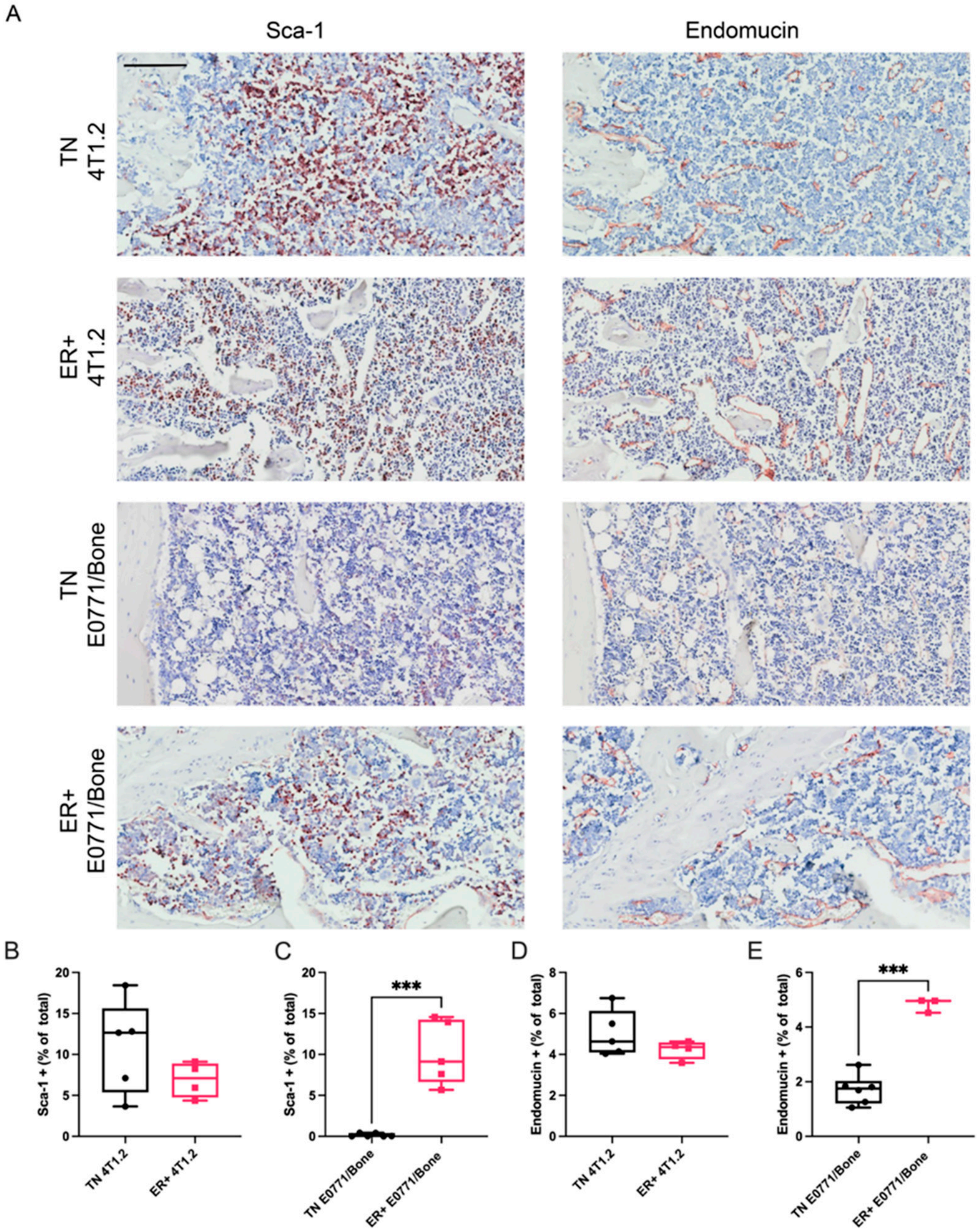

Supplemental Figure S8. Within the tibiae of mice with ER+ E0771/Bone tumors, there were increased HSCs and vasculature compared with mice with TN E0771/Bone-derived tumors. Immunohistochemistry for the HSC marker Sca-1 (A) was performed on tibiae from mice with ER+ and TN 4T1.2 and E0771/Bone tumors and was analyzed using a custom-made app on the pathology analysis software, VisioPharm (n=4–7, B and C). Scale bar represents 150 µm. There was a significant increase in percentage of Sca-1-positive area within the medullary cavity of the mice with ER+ E0771/Bone tumors when compared with mice with TN E0771/Bone tumors

(mean ± SEM, n=5–7, Student's t-test, *** represents p<0.005; C). Immunohistochemistry for the blood vessel marker endomucin (A) was performed on tibiae from mice with ER+ and TN 4T1.2 and E0771/Bone tumors and was analyzed using a custom-made app on the pathology analysis software, VisioPharm (n=4–7, D and E). There was a significant increase in percentage of endomucin-positive area within the medullary cavity of the mice with ER+ E0771/Bone tumors when compared with mice with TN E0771/Bone (mean ± SEM, n=3–7, Student's t-test, *** represents p<0.005; E).

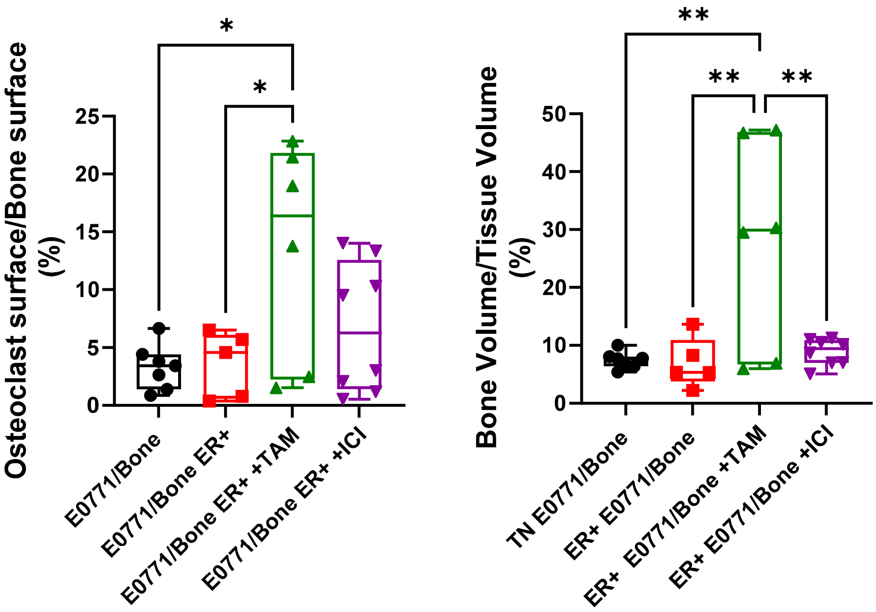

Supplemental Figure S9. TAM induced alterations in the bone niche. Tibiae were isolated from mice after tumor implantation with ER+ or TN E0771/Bone BC and treatment with vehicle controls, 5 mg/ 60-day time-release pellet TAM, or 1 mg/wk ICI. Bones were stained for TRAP+ osteoclasts and bone histomorphometry was performed to calculate bone volume to tissue volume fraction or osteoclast surface per bone surface. TAM treatment increased bone volume to tissue volume compared with all other groups. Mice with ER+ E0771/Bone tumors treated with TAM had significantly increased percentages of osteoclasts at the bone surface compared with ER+ E0717/bone without treatment and the E0771/Bone tumors. Both are represented as mean percentage ± SEM (n=4–8). * represents p<0.05 and ** represents p<0.01 by one-way ANOVA.
